# Combinatorial control of gene expression by the three yeast repressors Mig1, Mig2 and Mig3

**DOI:** 10.1186/1471-2164-9-601

**Published:** 2008-12-16

**Authors:** Jakub Orzechowski Westholm, Niklas Nordberg, Eva Murén, Adam Ameur, Jan Komorowski, Hans Ronne

**Affiliations:** 1 The Linnaeus Centre for Bioinformatics, Uppsala University, Box 598, SE-751 24 Uppsala, Sweden; 2 Department of Medical Biochemistry and Microbiology, Uppsala University, Box 582, SE-751 23 Uppsala, Sweden; 3 Interdisciplinary Centre for Mathematical and Computational Modelling, Warsaw University, 02-106 Warsaw, Poland

## Abstract

**Background:**

Expression of a large number of yeast genes is repressed by glucose. The zinc finger protein Mig1 is the main effector in glucose repression, but yeast also has two related proteins: Mig2 and Mig3. We have used microarrays to study global gene expression in all possible combinations of *mig1*, *mig2 *and *mig3 *deletion mutants.

**Results:**

Mig1 and Mig2 repress a largely overlapping set of genes on 2% glucose. Genes that are upregulated in a *mig1 mig2 *double mutant were grouped according to the contribution of Mig2. Most of them show partially redundant repression, with Mig1 being the major repressor, but some genes show complete redundancy, and some are repressed only by Mig1. Several redundantly repressed genes are involved in phosphate metabolism. The promoters of these genes are enriched for Pho4 sites, a novel GGGAGG motif, and a variant Mig1 site which is absent from genes repressed only by Mig1. Genes repressed only by Mig1 on 2% glucose include the hexose transporter gene *HXT4*, but Mig2 contributes to *HXT4 *repression on 10% glucose. *HXT6 *is one of the few genes that are more strongly repressed by Mig2. Mig3 does not seem to overlap in function with Mig1 and Mig2. Instead, Mig3 downregulates the *SIR2 *gene encoding a histone deacetylase involved in gene silencing and the control of aging.

**Conclusion:**

Mig2 fine-tunes glucose repression by targeting a subset of the Mig1-repressed genes, and by responding to higher glucose concentrations. Mig3 does not target the same genes as Mig1 and Mig2, but instead downregulates the *SIR2 *gene.

## Background

Gene regulatory networks control gene expression in response to both internal conditions (*e.g*. cell type, age) and external signals (*e.g*. nutrients, stress, signaling molecules). The use of combinations of transcription factors in regulatory networks greatly enhances the number of possible gene expression patterns, and enables cells to fine-tune their response to different conditions. Combinatorial aspects of gene regulation have been studied both on a whole-network scale [1-6] and for specific parts of regulatory networks [7-9]. In the present study, we examine combinatorial gene regulation during glucose repression in the budding yeast *Saccharomyces cerevisiae*.

Glucose is the preferred carbon source for *S. cerevisiae*, which metabolizes glucose by a purely glycolytic process (fermentation) even under aerobic conditions. Fermentation produces fewer moles of ATP per mole of glucose than oxidation to CO_2 _and H_2_O, but the rate of ATP production is higher, enabling faster growth [10]. During fermentative growth, NADH is regenerated by reducing the pyruvate that is formed to ethanol, which is then exported from the cell. Once the glucose has been consumed, the cell switches to aerobic metabolism of the ethanol. This switch is called the diauxic shift.

The difference between fermentative and aerobic growth is in part mediated by a regulatory mechanism called glucose repression [10-13]. Glucose repressed genes can be divided into three groups. The first includes genes needed for uptake and metabolism of other carbon sources, such as galactose, maltose, and ethanol. Glucose repression of these genes overrides specific induction, such as galactose induction of the *GAL *genes. The second group includes genes needed for the Krebs cycle and oxidative phosphorylation, which are dispensable during fermentative growth. The third group of glucose repressed genes are those involved in gluconeogenesis, which need to be repressed on glucose to prevent futile cycling.

**Figure 1:**
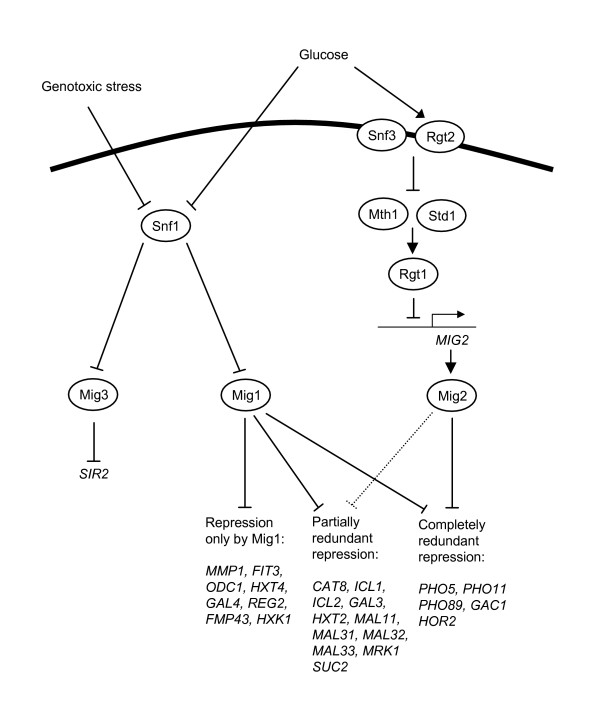
**Overview of the glucose repression and glucose induction pathways in the yeast *S. cerevisiae***. For each regulatory step, positive control is shown as an arrow, and negative control as a crossbar. Examples of differentially regulated genes that are discussed in the present study are shown at the bottom.

The main effector in glucose repression is Mig1 (Figure 1), a zinc finger protein that binds to the promoters of many genes and represses their transcription [14-18]. Repression by Mig1 can be both direct and indirect, through repression of genes encoding transcriptional activators. One example of this are the *GAL *genes, which are repressed both directly by Mig1, and indirectly through repression of the *GAL4 *gene [16]. Yeast has two other zinc finger proteins that are closely related to Mig1: Mig2 and Mig3 [19,20]. Mig2 seems to be a minor player in glucose repression. Some glucose repressed genes are synergistically repressed by Mig1 and Mig2 while others are repressed only by Mig1. No genes have so far been shown to be repressed only by Mig2. Mig3 has been reported to contribute marginally to glucose repression of some genes [19,21].

Mig1 is negatively regulated by Snf1, a protein kinase present in all eukaryotes (Figure 1). Snf1 is called the AMP-activated protein kinase in animals, where it is regulated by AMP. This kinase has a general function in energy homeostasis [22]. It is activated at low energy conditions and restores the energy level by stimulating energy producing processes and inhibiting energy consuming processes. Yeast Snf1 is inhibited in the presence of glucose, which can be regarded as a high energy condition. In the absence of glucose, Snf1 phosphorylates Mig1. This causes nuclear exclusion of Mig1 [23] and prevents its interaction with the Cyc8-Tup1 co-repressor [24], thereby causing derepression of glucose repressed genes. Glucose repression thus involves three consecutive negative steps: glucose inhibits Snf1, which inhibits Mig1, which represses target genes [23,25,26].

There is also a glucose induction pathway in yeast, which activates genes required for uptake and metabolism of glucose (Figure 1). It works through the two glucose sensors Snf3 and Rgt2 [10]. In the presence of glucose, Snf3 and Rgt2 generate a signal that stimulates degradation of Mth1 and Std1, two proteins that are required for expression of Rgt1 [27]. Rgt1 is a Gal4-related zinc cluster protein that represses the glucose induced genes [21,28]. The end result is therefore that these genes are derepressed, *i.e*. induced, in the presence of glucose.

The nuclear localization of Mig2 is not controlled by glucose, and there is no evidence that Mig2 is regulated by Snf1. Instead, the *MIG2 *gene is induced by glucose [21]. Mig3 is under dual level control by glucose. Thus, the *MIG3 *gene is glucose induced [21], and the Mig3 protein is subject to Snf1-dependent phosphorylation and subsequent degradation in the absence of glucose [29]. The *MIG3 *gene is also induced by genotoxic stress, and there is evidence that Mig3 functions as a downstream effector in the Snf1-dependent response to hydroxyurea [29]. Finally, there is cross-talk between the glucose repression and glucose induction pathways. Thus, *SNF3 *and *MTH1 *are repressed by Mig1, and *MIG1 *is repressed by Mig2 [21].

Mig1, Mig2 and Mig3 have similar DNA-binding zinc fingers, and the residues thought to be important for the DNA specificity [15] are conserved. The site bound by Mig1 is well characterized and consists of a GC-rich core, (C/G)(C/T)GG(G/A)G (referred to as a Mig1 site below), and an AT-rich region 5' to the GC-box [15]. No mismatches are allowed within the GC-box, but one or two C's or G's are tolerated within the AT-box [15]. Mig2 and Mig3 have both been shown to bind to some Mig1 sites *in vitro *[19,20], but the precise specificity of either protein has not yet been determined.

Several previous studies have investigated the roles of Mig1, Mig2 and Mig3 in gene expression. The expression of five reporter genes were examined in different mutants using β-galactosidase fusions [21]. Filter hybridizations were used to compare expression of a subset of the yeast transcriptome in a *mig1 mig2 mig3 *triple mutant and the wild type, after which β-galactosidase fusions were used to further study the expression of 25 genes [19]. A genome-wide study that identified promoter regions bound by different transcription factors included data for Mig1, Mig2 and Mig3 [30]. Finally, microarrays were used to study gene expression in several mutants affecting glucose signaling, including *mig1 *and *mig1 mig2 *mutants [31]. None of these previous studies has provided a comprehensive picture of how the expression of the entire yeast transcriptome is affected by Mig1, Mig2, and Mig3. We have now undertaken such a comprehensive study in order to elucidate combinatorial effects in glucose repression, and to better understand the different roles played by Mig1, Mig2 and Mig3.

## Results

In order to elucidate how the three related yeast repressors Mig1, Mig2 and Mig3 contribute to the control of gene expression, we used microarrays to study the expression of all yeast genes in the wild type and all seven possible combinations of *mig1, mig2 *and *mig3 *deletions. Since Mig1 and Mig2 both are involved in glucose repression, the experiments were carried out in the presence of 2% glucose. Each experiment was performed at least in triplicate, with independent biological samples. To facilitate the analysis, and to reduce the number of false positives, we used the following criteria for which genes were further considered: genes whose average expression went up or down at least 2-fold in at least one comparison between different mutants, and where the effect was significant at p < 0.05 when comparing data from replicate arrays.

### Partial redundancy of Mig1 and Mig2, but not Mig3, in gene regulation

**Figure 2:**
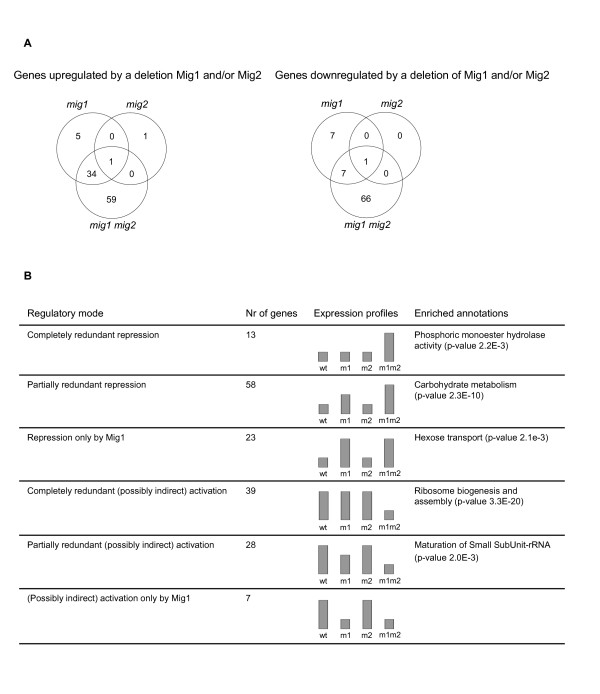
**Combinatorial effects of Mig1 and Mig2 on gene expression**. A) Number of genes that are either upregulated or downregulated in the *mig1*, *mig2 *and *mig1 mig2 *strains, as compared to the wild type. B) Six different ways in which Mig1 and Mig2 can cooperate in regulating target genes. Both upregulated and downregulated genes were divided into three groups depending on their redundancy measures. For each set of genes the following information is shown: the number of genes, the typical expression profile (in the wild type, *mig1*, *mig2*, and *mig1 mig2 *strains), and enriched Gene Ontology annotations, if any.

Using these criteria, we found that 100 genes are upregulated and 81 genes are downregulated in response to a deletion of Mig1 and/or Mig2, as compared to the wild type (Figure 2A). These genes are listed in additional file 1: Table S1 and additional file 2: Table S2. Of these, 94 genes were upregulated and 74 genes were downregulated in the *mig1 mig2 *double mutant. In the *mig1 *single mutant, 40 genes were upregulated and 15 genes downregulated. As expected, most of these genes are included in the larger set of genes that are affected in the double mutant (Figure 2A). To quantify the redundancy in regulation by Mig1 and Mig2, we calculated a redundancy measure, *r*, which is 1 if a gene shows complete redundancy in its dependence on Mig1 and Mig2 (*i.e*. if only the *mig1 mig2 *double deletion has an effect, with no effect of the *mig1 *single deletion), and 0 if a gene depends only on Mig1 (*i.e*. if the *mig1 *single deletion has the same effect as the *mig1 mig2 *double deletion). Another way to interpret *r *is as a measure of the contribution of Mig2, with 1 indicating that Mig2 can fully control expression of the gene in the absence of Mig1, and 0 indicating that Mig2 does not at all contribute to its regulation. The genes were then divided into three sets: genes with *r *≥ 0.75 were considered as completely redundantly regulated, those with 0.25 ≤ *r *< 0.75 as partially redundantly regulated, and those with *r *< 0.25 as being regulated by Mig1 alone.

As seen in Figure 2B, we found that of the 94 genes that are upregulated in the *mig1 mig2 *double mutant, 71 show either complete or partial redundancy in their dependency on Mig1 and Mig2 while 23 genes are regulated by Mig1 alone. This is consistent with previous findings that while some glucose repressed genes are repressed by Mig1 alone, most depend on both Mig1 and Mig2 [19,21,31]. For activated genes, *i. e*. genes that are downregulated in the mutants, we saw a similar pattern, with 67 of 74 genes showing complete or partial redundancy for Mig1 and Mig2, and 7 genes being affected only by Mig1 (Figure 2B).

In the *mig2 *single mutant, only two genes were upregulated using our criteria, but they failed to appear in other comparisons involving *mig2*. However, *HXT6*, which was a borderline case (upregulated 1.97-fold in the *mig1 mig2 *mutant) showed a smaller (1.72-fold) but still significant increase in the *mig2 *single mutant. Further studies using real-time PCR confirmed that *HXT6 *is upregulated in *mig2 *strains (see below). A deletion of *MIG3 *had very limited effects in our experiment. Only four genes were upregulated more than 2-fold, and only one of them, *MRK1*, has a Mig1 site in its promoter and is also upregulated in the *mig1 mig2 *double mutant. Nor did a deletion of *MIG3 *in the *mig1 *and *mig2 *single or double deletion mutants have any further effects. As discussed below, the *SIR2 *gene is upregulated in the *mig3 *mutant, but *SIR2 *is not a target for Mig1 or Mig2. We conclude that Mig3 does not contribute much to the regulation of the genes that are regulated by Mig1 and Mig2, at least not on 2% glucose.

### The degree of Mig1/Mig2 redundancy correlates with gene classification

Interestingly, we found that the degree of redundancy (*i.e*. the contribution of Mig2) is strongly correlated with certain functional annotations. This is particularly true for genes that are upregulated in the mutants (Figure 2B). Since most of these genes have Mig1 binding sites in their promoters (see below), we consider them to be likely direct targets of Mig1/Mig2-dependent repression. Genes that are repressed only by Mig1 (*e.g*. *MTH1*, *HXK1 *and *HXT4*) are enriched for the annotation *hexose transport*. Moreover, several other genes in this group (*e.g. ODC1*, *FIT3 *and *MMP1*) are involved in other kinds of transport. Genes under partially redundant repression are enriched for the annotation *carbohydrate metabolism*, and many previously known glucose repressed genes fall into this category. The group of genes that are completely redundantly repressed by Mig1 and Mig2, finally, includes genes involved in phosphate metabolism (*PHO5, PHO11 *and *PHO89*) and is therefore enriched for the annotation *phosphoric monoester hydrolase activity*. *PHO89 *was the most strongly affected gene in the yeast genome, being upregulated 56-fold in the *mig1 mig2 *double mutant, whereas *PHO5 *and *PHO11 *had the highest redundancy measures of all genes (see additional file 1: Table S2). For genes that depend on Mig1 and/or Mig1 for activation, *i. e*. are downregulated in the mutants (Figure 2B), we found that redundant regulation is strongly correlated with the annotation *ribosome biosynthesis and assembly*, and that partially redundant regulation correlates with *maturation of small subunit rRNA*. No annotation was significantly enriched among the seven genes that depend on Mig1 only.

### Mig1 sites and STRE motifs are both enriched in promoters that are repressed by Mig1 and/or Mig2

As expected, the promoter regions of all genes that are repressed by Mig1 and/or Mig2 are enriched for the Mig1 site (CG)(CT)GG(G/A)G (p-value 1.5e-28). In total, 96% of the repressed promoters contained at least one Mig1 site, as compared to 41% of all yeast promoters. Surprisingly, we found that these promoters are also enriched for the STRE element AGGGG (p-value 5.7e-8). The STRE element is also a GC-rich sequence, but it does not bind Mig1 or Mig2. Instead, it is the target of four other zinc finger proteins, Msn2, Msn4, Gis1 and Rph1, which are involved in various types of stress signalling [32-34]. It should be noted that STRE elements are more common in promoters that also contain Mig1 sites. We therefore tested if this correlation can explain the enrichment of STRE elements in promoters that are repressed by Mig1 and/or Mig2. Our analysis suggests that this is indeed the case.

It is not clear why Mig1 sites and STRE motifs occur in the same promoters. However, we note that many genes that contain STRE motifs are upregulated during the diauxic shift, when glucose is depleted [35], so dual control of these genes by glucose repression and stress signalling is not unexpected. Genes that contain the PDS element, a third motif through which Gis1 and Rph1 act, are also upregulated during the diauxic shift [34,36,37]. However, unlike the STRE elements, we did not find any significant enrichment of PDS elements in the promoters of genes that are repressed by Mig1 and/or Mig2, nor is the PDS element overrepresented in genes that contain Mig1 sites (data not shown).

### Nucleosome displacement and nucleosome phasing at functional Mig1 sites

It is not unusual for promoters to contain cryptic binding sites for transcription factors, that are not used *in vivo *due to an unfavourable position or context. Consistent with this, many genes with Mig1 sites in their promoters are not significantly upregulated in the *mig1 mig2 *double mutant. We wished to determine if there is a difference in nucleosome occupancy at Mig1 sites in promoters that are regulated by Mig1 and/or Mig2 and those that are not, which would support the notion that the latter contain cryptic rather than functional Mig1 sites. We therefore used nucleosome occupancy data [38] to examine nucleosome positioning around Mig1 sites in both sets of genes. As a control, we used the STRE element, which as discussed above also is enriched in the promoters of genes that are regulated by Mig1 and/or Mig2.

**Figure 3:**
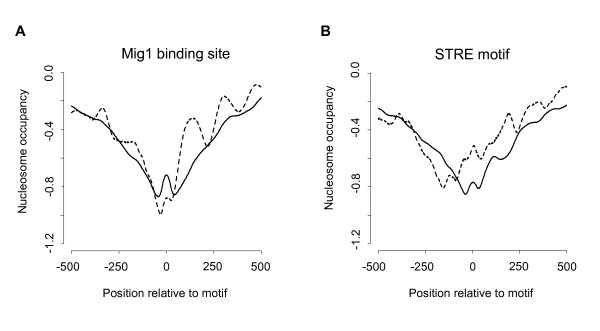
**Nucleosome occupancy around Mig1 sites and STRE elements**. Nucleosome occupancies around A) Mig1 sites and B) STRE elements in promoters that are repressed by Mig1 and/or Mig2 on 2% glucose are shown by dashed lines, and occupancies around the same motifs in other promoters by solid lines. The occupancy data was from Lee et al. (2007).

Promoters generally contain fewer nucleosomes, which is evident from the V-shaped troughs in all nucleosome occupancy plots in Figure 3. However, as shown in Figure 3A, we found that nucleosome occupancy at Mig1 sites is lower in promoters of Mig1/2-repressed genes than in other promoters with Mig1 sites (Wilcoxon rank-sum-test p-value 1.5e-3). Since nucleosomes and transcription factors frequently prevent each other from accessing DNA, the reduced nucleosome occupancy at Mig1 sites in Mig1/2-repressed genes is a further validation that these sites are functional. For the STRE elements, we saw a different pattern (Figure 3B). Thus, the nucleosome occupancy at STRE elements is higher in promoters of Mig1/2-repressed genes than in other promoters (Wilcoxon rank-sum-test p-value 6.5e-3). These results support the notion that the Mig1 sites, but not the STRE elements, are important for repression mediated by Mig1 and Mig2.

Interestingly, we further found that nucleosome occupancy downstream of functional Mig1 sites is clearly phased, with an average distance between peaks of 165 base pairs (Figure 3A). In contrast, no clear phasing was seen upstream of functional Mig1 sites, around cryptic Mig1 sites (Figure 3A), or around the STRE elements in either sets of genes (Figure 3B). We conclude that this nucleosome phasing is specific for functional Mig1 sites. It suggests that nucleosomes bind in an ordered fashion downstream, but not upstream, of functional Mig1 sites.

### Redundantly downregulated promoters are enriched for Pho4 binding sites and a novel GGGAGG motif

We proceeded to study how the specificity of the combinatorial regulation is achieved, *i.e*. what makes downregulation of some genes depend only on Mig1 whereas other genes depend redundantly on both Mig1 and Mig2. We examined six possible hypotheses: a) *variations in the sequence of the Mig1/2 site *account for different binding affinities of Mig1 and Mig2; b) *the importance of the flanking AT box for binding *differs between Mig1 and Mig2; c) sites at *different positions *in the promoter may be differentially recognized by Mig1 or Mig2; d) Mig1 and Mig2 have preferences for *different orientations *of the binding sites; e) the number of Mig1 sites is important; and f) *other DNA motifs *are involved.

**Figure 4:**
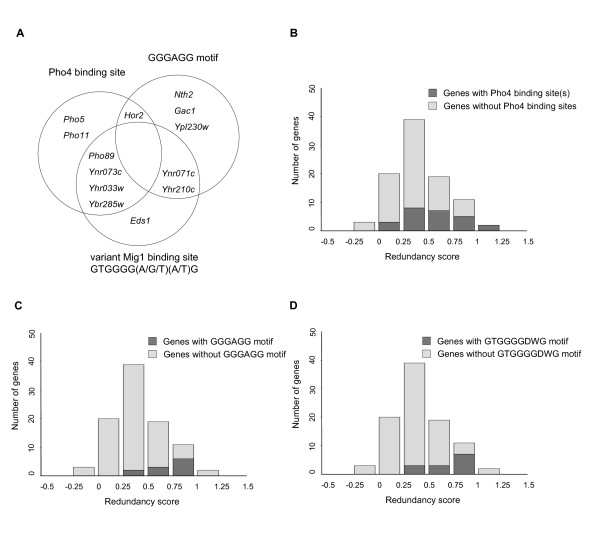
**Enrichment of sequence motifs in promoters that are redundantly repressed by Mig1 and Mig2**. A) Presence or absence of the Pho4 binding site [CACGT(G/T)], the novel GGGAGG motif, and the variant Mig1 site [GTGGGG(A/G/T)(A/T)G] in the 13 most redundantly repressed genes. B) Relative enrichment of the Pho4 binding site in the promoters of all redundantly repressed genes. C) Relative enrichment of the GGGAGG motif in the promoters of all redundantly repressed genes. D) Relative enrichment of the variant Mig1 binding site in the promoters of all redundantly repressed genes. The histograms show the number of promoters with and without at least one motif for different values on the redundancy measure. The differences between genes with and without motifs were tested for significance using Wilcoxons rank-sum test.

The only two hypotheses for which we found strong support were a) and f). Starting with the latter, we found that two other DNA motifs, the Pho4 binding site CACGT(G/T) and the motif GGGAGG, are significantly overrepresented in genes that are redundantly repressed by Mig1 and Mig2 (p-values of 3.7e-3 and 2.2e-4, respectively) (Figure 4A). The presence of Pho4 sites in this set of genes is consistent with our finding that it is enriched for genes annotated for *phosphoric monoester hydrolase activity*. It is not known what yeast transcription factors, if any, bind to the GGGAGG motif, nor has it previously been associated with a specific group of genes. The single gene that had neither a Pho4 binding site nor a GGGAGG motif, *EDS1*, encodes a zinc cluster protein with unknown function. However, the closest relative of Eds1 is the Rgt1 repressor involved in glucose induction. This raises the possibility that Eds1 also is involved in glucose control of gene expression, which could explain its unusal mode of repression by Mig1 and Mig2.

### Redundantly repressed promoters are enriched for a variant Mig1 site which is absent from promoters repressed only by Mig1

As stated above, we also found strong support for hypothesis a), *i.e*. that the sequence of the Mig1 site is important for the degree of redundant repression. Thus, using the BCRANK program (see Methods), we found that a variant of the Mig1 site, GTGGGG(A/G/T)(A/T)G, is significantly enriched (p-value 9.4e-5) in the promoters of the redundantly repressed genes (Figure 4A). In total, 7 of the 13 redundantly repressed genes, including *EDS1*, have the GTGGGG(A/G/T)(A/T)G motif in their promoters. Interestingly, we further noted that the 23 genes that are repressed only by Mig1 do not contain a single such variant Mig1 site. This prompted us to further test the hypothesis that this motif is important for redundancy by looking at the fraction of GTGGGG(A/G/T)(A/T)G motifs among all Mig1 sites in the three groups of genes. As shown in Table 1, the variant motif accounts for 8 of 37 Mig1 sites in the promoters of the redundantly repressed genes, which is a significant overrepresentation (p-value 4.1e-4). Among promoters that are repressed only by Mig1, the motif is found at 0 of 55 Mig1 sites, which is a significant underrepresentation (p-value 1.6e-2). Promoters that show partially redundant repression by Mig1 and Mig2 do not differ significantly from the expected number of variant motifs.

**Table 1:** Distribution of the variant Mig1 motif among genes repressed by Mig1 and/or Mig2

Gene group	Variant/Total *	p-value
Completely redundant repression	8/37	4.1e-4 **
Partially redundant repression	6/130	1.7e-1 **
Repression only by Mig1	0/55	1.6e-2 ***
All Mig1/2 repressed genes	14/222	

* Fraction of variant Mig1 sites [GTGGGG(A/G/T)(A/T)G] out of all Mig1 sites [(CG)(CT)GG(GA)G]

** Significance test for over-representation of variant Mig1 sites

*** Significance test for under-representation of variant Mig1 sites

### Promoters whose activation depend on Mig1 and/or Mig2 are enriched for PAC and rRPE motifs

Some of the genes that are downregulated in the *mig1 mig2 *double mutant have Mig1 sites in their promoters (see additional file 2: Table S2), but these sites are not significantly enriched in this group of genes. This suggests that most of these genes are not direct targets for activation by Mig1 or Mig2. We therefore considered the possibility that some of them could be regulated indirectly, by a repressor that is glucose repressed. One obvious example are the two low affinity hexose transporters *HXT1 *and *HXT3*, that are known to be regulated by the Rgt1 repressor. However, Rgt1 sites are not enriched among all promoters that depend on Mig1 and/or Mig2. Instead, we found that two other motifs are significantly enriched among these promoters: the PAC motif (p-value 8.7e-13) and the rRPE site (p-value 4.2e-7). These two motifs are known to be involved in regulation of rRNA processing genes [39], which is consistent with the annotations *ribosome biogenesis and assembly *and *maturation of small subunit rRNA *being enriched among redundantly activated genes (Figure 2B). The rRPE site is bound by Stb3 which activates transcription in response to glucose [40], but expression of Stb3 was not affected by deletions of Mig1 or Mig2 in our microarray experiments (data not shown). It is not known which transcription factors, if any, bind to the PAC site.

### Combinatorial regulation of the *HXT *genes by Mig1 and Mig2

**Figure 5:**
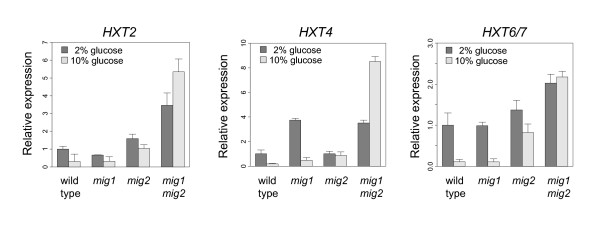
**Real-time PCR quantifications of *HXT *gene expression**. Real-time PCR quantifications of *HXT *mRNA in wild type, *mig1*, *mig2*, and *mig1 mig2 *cells grown on both 2% and 10% glucose. All values were normalized relative to the expression in the wild type strain on 2% glucose. The plot shows mean values of three biological replicates, with standard deviations indicated by the error bars.

The *HXT *genes are interesting for two reasons: they are subject to both glucose induction and glucose repression, and different *HXT *genes respond differentially to different levels of glucose [41,42]. Our finding that *HXT6 *is one of the few genes whose expression is more sensitive to Mig2 prompted us to examine the expression of the *HXT *genes in detail, using real-time PCR and two different glucose concentrations. Thus, we measured the expression of *HXT2, HXT4 *and *HXT6/7 *in the *mig1*, *mig2 *and *mig1 mig2 *strains grown on either 2% or 10% glucose (Figure 5).

We found that *HXT2 *is redundantly repressed by Mig1 and Mig2 on 2% glucose. This is in agreement with earlier findings [20,28,42] and with our microarray data in (see additional file 3: Figure S1). On 10% glucose, there was a similar but stronger effect, especially in the *mig2 *and *mig1 mig2 *strains. This suggests that Mig2 is more important on 10% glucose than on 2% glucose. The *HXT4 *gene was only repressed by Mig1 on 2% glucose. Again, this is in agreement with earlier results [28,31] and with our microarray data. On 10% glucose, however, *HXT4 *was repressed by both Mig1 and Mig2, with Mig2 seemingly being the stronger repressor. It was even more so than for *HXT2*; it therefore seems that Mig2 is more important for repression on 10% glucose than on 2% glucose.

The high affinity transporter genes *HXT6 *and *HXT7 *are highly homologous, differing in only three nucleotide positions. This makes it very hard to distinguish between the two transcripts when measuring gene expression. We were therefore only able to measure the combined expression of *HXT6 *and *HXT7 *in our real-time PCRs, and it is likely that the microarray data for either gene also is the sum of the expression of the two genes. This is not a serious drawback since all previous evidence suggests that these two duplicated genes are identically regulated, but the caveat must be added that we could not tell them apart.

Interestingly, *HXT6/7 *is one of the few cases where Mig2 seems to be more important for repression than Mig1 on 2% glucose. Thus, we saw a small (1.72-fold) but significant increase in *HXT6/7 *expression in the *mig2 *strain as compared to the wild type, and a further increase (2-fold) in the *mig1 mig2 *strain, which is consistent with the microarray data in additional file 3: Figure S1. Also consistent with the latter, we saw no difference in expression in the *mig1 *strain. On 10% glucose the pattern was even more pronounced, with a 7-fold increase in the *mig2 *strain as compared to the wild type, a 19-fold increase in the *mig1 mig2 *strain and no significant effect in the *mig1 *strain. Thus, *HXT6/7 *seem to be regulated by both Mig1 and Mig2, with Mig2 being the main repressor. This is the case both on 2% and on 10% glucose, but again the effect of the *mig2 *deletion is much stronger on 10% glucose.

Expression of the *HXT1 *and *HXT3 *genes encoding the two low affinity transporters was only studied in the microarray experiment, *i. e*. on 2% glucose. It has previously been reported that *HXT1 *is downregulated in response to a deletion of *MIG1 *and *MIG2 *[19]. We saw the same effect. Thus, *HXT1 *was downregulated to 4% of the wild type in the *mig1 mig2 *strain, and no effects were seen in single mutants (see additional file 3: Figure S1,). This suggests that Mig1 and Mig2 function redundantly in their positive effect on *HXT1 *expression. The *HXT3 *gene, finally, was slightly upregulated in the *mig1 *mutant (1.3-fold, but significant at p = 0.034) and downregulated in the *mig1 mig2 *double mutant (to 34% of the wild type value).

### The *SIR2 *gene is downregulated by Mig3

**Figure 6:**
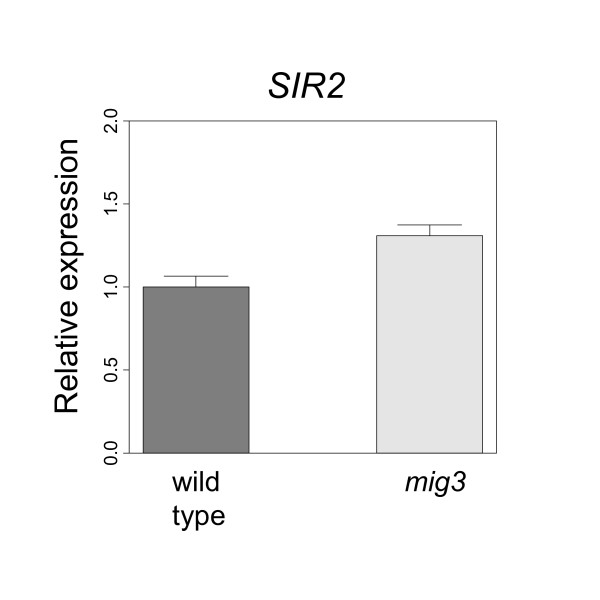
**Real-time PCR quantification of *SIR2 *gene expression**. Real-time PCR quantification of *SIR2 *mRNA in wild type and *mig3 *cells grown on 2% glucose. Values were normalized relative to the expression in the wild type strain. The plot shows mean values of five biological replicates, with standard deviations indicated by the error bars.

As noted above, we found no evidence that Mig3 regulates the same set of genes as Mig1 and Mig2 on 2% glucose. In fact, only one of the genes in Figure 2, *MRK1*, was upregulated in the *mig3 *deletion. Moreover, the four genes that were upregulated more than 2-fold in the *mig3 *deletion (*CyB*, *AGA1*, *INO2 *and *MRK1*) were not significantly upregulated in the *mig3 *double and triple deletions. However, when we included genes that are upregulated more than 1.5-fold in the *mig3 *deletion, we found one gene which fulfills both criteria: a low p-value (2.5e-3) and a significant upregulation also in the *mig1 mig3*, *mig2 mig3 *and *mig1 mig2 mig3 *strains relative to the wild type. This was the *SIR2 *gene, which encodes a histone deacetylase involved in silencing. The effect of the *mig3 *deletion on *SIR2 *expression was a 1.69 fold increase. To confirm that *SIR2 *is downregulated by Mig3, we proceeded to measure the expression of *SIR2 *by real time PCR. Again, we saw a small (1.31 fold) but significant (p-value 1.2e-3) increase in *SIR2 *expression in the *mig3 *strain (Figure 6). The mechanism behind this regulation remains to be determined, but we note that the *SIR2 *promoter does not contain a Mig1 site.

## Discussion

We have used microarrays to study gene regulation by the three yeast zinc finger proteins Mig1, Mig2 and Mig3. In particular, we wanted to investigate to what extent they cooperate in gene regulation, and whether such cooperation is redundant or synergistic. To this end, we studied the expression of all yeast genes in all possible combinations of *mig1*, *mig2 *and *mig3 *deletion mutants. Consistent with previous studies [19-21,31], we found that Mig1 and Mig2 regulate an overlapping set of genes on 2% glucose (see additional file 1: Table S1 and additional file 2: Table S2), with Mig1 being the most important regulator. Thus, some genes depend only on Mig1, other genes are regulated both by Mig1 and Mig2, but very little effect is seen when only Mig2 is deleted (Figure 2A).

Interestingly, we further found that the degree of redundancy in regulation by Mig1 and Mig2 correlates with the gene ontology classification, indicating that genes involved in the same type of processes show a similar type of regulation (Figure 2B). This was particularly evident for repressed genes, *i.e*. genes that are upregulated in the *mig1 mig2 *double mutant. Thus, genes that are repressed only by Mig1 tend to be involved in transport (in particular hexose transport), genes that are repressed mostly by Mig1 but also to some extent by Mig2 are involved in carbohydrate metabolism and genes that are repressed redundantly by Mig1 and Mig2 tend to be involved in phosphate metabolism. We further note that most of the genes that are repressed by Mig1 and/or Mig2 are expressed after glucose depletion [35], which is consistent with them being controlled by glucose repression.

Our finding that a variant Mig1 site, GTGGGG(A/G/T)(A/T)G, is significantly enriched in redundantly repressed promoters, but absent from promoters repressed only by Mig1, suggests that differences in sequence specificity between Mig1 and Mig2 may account for different modes of regulation. According to this model, Mig2 would have a narrower sequence specificity than Mig1. Furthermore, genes that are redundantly repressed would contain Mig1 sites that can also bind Mig2, while genes repressed only by Mig1 would lack such sites. Our results suggest that GTGGGG(A/G/T)(A/T)G may, indeed, be one such variant motif capable of binding Mig2, but it cannot be the only one, since several redundantly repressed genes lack this motif. Further experiments are needed to test this hypothesis and to determine the precise binding specificity of Mig2.

In addition to the GTGGGG(A/G/T)(A/T)G motif, we found that the redundantly repressed promoters are enriched for two other motifs: the Pho4 binding site [CACGT(G/T)], and the novel GGGAGG motif. Pho4 is a downstream effector in the Pho pathway, which activates genes encoding phosphatases (*PHO5*, *PHO8*, *PHO11*, and *PHO12*), phosphate transporters (*PHO84 *and *PHO89*) and other genes involved in phosphate utilization in response to phosphate starvation [43-45]. Our finding that redundantly repressed genes are enriched for Pho4 binding sites is therefore consistent with our finding that these genes tend to be involved in phosphate metabolism. We interpret this as correlation rather than causation, *i. e*. the Pho4 sites are found in these genes because they are involved in phosphate metabolism, and not because Pho4, directly or indirectly, contributes to the redundant mode of repression. Consistent with this, expression of Pho4 is not affected by deletion of Mig1 or Mig2, neither is expression of components in the Pho pathway such as Pho2, Pho80, Pho85 or Pho81 (data not shown). Also, there is no significant depletion of nucleosomes around Pho4 sites in the promoters of the redundantly repressed genes, compared to Pho4 sites in all other promoters (data not shown).

The GGGAGG motif has not previously been described in yeast, and it is not known which proteins, if any, bind to it. The mammalian zinc finger proteins Sp1 and Sp3 bind to this motif (and other similar motifs) [46,47], but the DNA specificity of the GC-box binding zinc finger proteins (to which Mig1, Mig2, Sp1 and Sp3 belong) is determined by amino acid residue 18 in each finger [15], and there is no reason to expect that Mig1 or Mig2 would bind to this motif. We further make three observations. Firstly, among the 13 redundantly repressed genes, those containing Pho4 binding sites (7 genes) and the GGGAGG motif (6 genes) are largely complementary (only one gene has both motifs). Secondly, these two groups cover 12 of the 13 genes. Thirdly, genes with the GGGAGG motif include *NTH2 *encoding a trehalase, *HOR2 *encoding a glycerol-3-phosphatase, and *GAC1 *encoding a protein phosphatase 1 regulatory subunit. These proteins are all directly or indirectly connected to phosphate metabolism. We conclude that most of the redundantly repressed genes are involved in phosphate metabolism, and usually contain either Pho4 binding sites or GGGAGG motifs, but rarely both. This suggests that the GGGAGG motif may bind a transcription factor that regulates genes involved in phosphate metabolism that are not directly regulated by Pho4.

The physiological reason for this unexpected link between glucose repression and phosphate metabolism remains to be determined. One connection found in the literature is the fact that a deletion of Snf1 causes a defect in glycogen accumulation, which can be suppressed by mutations in Pho85 [48], suggesting that Pho85 may be involved in mediating regulation of glycogen by Snf1. Furthermore, it has been shown that the *GSY2 *gene encoding glycogen synthase is regulated both by Mig1 and Pho85 [49]. There are also other, more general connections between glucose and phosphate signalling in yeast. Thus, it has been shown that addition of phosphate to phosphate starved cells affects a number of known targets of the PKA pathway, and that this effect is dependent on the presence of glucose and requires at least one of the two glucose sensing systems in the PKA pathway [50]. Finally, glucose depletion causes degradation of the Pho84 phosphate transporter [51].

At first sight, the fact that genes involved in phosphate uptake and salvage are repressed by Mig1 and Mig2 seems counter-intuitive, since phosphate is required for glucose metabolism. Furthermore, *PHO5 *and *PHO11 *are both downregulated when glucose is depleted [35], where one would instead expect Mig1/2-repressed genes to be upregulated. One possible explanation for *PHO5 *and *PHO11 *being downregulated after the diauxic shift is that they also are regulated by other signaling pathways that turn off their expression whether or not glucose is present. In particular, phosphate limitation comes to mind. However, we note that *PHO5 *and *PHO11 *are more highly expressed on 2% glucose also during logarithmic growth [52]. The question then remains why *PHO5 *and *PHO11 *are upregulated in the *mig1 mig2 *double mutant. It is possible that this reflects a repression at high glucose concentrations, which is partially active on 2% glucose, and that the downregulation in the absence of glucose is due to these genes also being glucose induced. However, it is also possible that Mig1 and Mig2 repress *PHO5 *and *PHO11 *in response to some other signal than glucose.

For *PHO89*, its upregulation in the *mig1 mig2 *double mutant is consistent with the fact that it is downregulated by 2% glucose and upregulated after glucose depletion [35,52]. What is intriguing in this case is the fact that *PHO89 *shows the strongest response of all yeast genes to loss of both Mig1 and Mig2: a 56-fold upregulation (see additional file 1: Table S1). For comparison, *SUC2*, a classical target of glucose repression, is upregulated only 21-fold. It should further be noted that this effect is much greater than the 3-fold difference seen in *PHO89 *expression in the presence or in the absence of glucose [52]. This again raises the question whether the effect of the *mig1 mig2 *double deletion just reflects loss of glucose repression, or whether Mig1 and Mig2 regulate *PHO89 *is response to some other signal than glucose. We note that *PHO89 *is known to be induced by cell wall damage in addition to being regulated by phosphate [53].

Interestingly, we found that for the *HXT *genes, the relative importance of Mig1 and Mig2 as repressors depends on the glucose concentration. At 2% glucose Mig1 is the major repressor, and the effects of a *mig2 *deletion are seen only in the absence of Mig1. On 10% glucose, Mig2 is more important. The clearest example of this is repression of *HXT4*, which depends only on Mig1 on 2% glucose, but on both Mig1 and Mig2 on 10% glucose (Figure 5). The same trend, with Mig2 being more important on 10% glucose, is also seen for *HXT2 *and *HXT6/7 *(Figure 5). If this result can be extended to other glucose repressed genes, it may explain why Mig2 has seemed to be of minor importance in previous studies [19,21]. It should be noted that the natural habitat of yeast, fermenting fruits and grapes, contains high concentrations of glucose. Mig2 could thus be more important under these conditions than under standard laboratory conditions. However, we note that differential repression makes sense for the *HXT *genes, where transporters with different affinities need to be expressed at different glucose concentrations. It remains to be seen how important differential regulation by Mig1 and Mig2 is for other glucose repressed genes.

It should be emphasized that the fact that the genes repressed only by Mig1 are enriched for the annotation *hexose transport *does not mean that all *HXT *genes are regulated in this way. In fact, the *HXT *genes respond differently to different glucose concentrations and to deletions of *mig1 *and *mig2*, consistent with the fact that they encode transporters with different affinities that are needed at different glucose concentrations. Thus, the high affinity transporter genes *HXT6 *and *HXT7 *are repressed only at high glucose concentrations, and it therefore makes sense that they are among the few genes that are more sensitive to Mig2. Furthermore, we note that whereas the *HXT4 *gene is repressed only by Mig1 on 2% glucose, Mig2 contributes significantly to its repression on 10% glucose. This could mean that the above discussed binding site specificities are relative rather than absolute, and that Mig2 may recognize all Mig1 sites, though with different affinities. In conclusion, the use of Mig1 and Mig2 would enable yeast cells to fine-tune their response to different concentrations of glucose.

The reason why Mig1 and Mig2 respond to different glucose levels in regulating the *HXT *genes remains to be determined: Activation of Mig1 by glucose involves inhibition of Snf1-dependent phosporylation of Mig1 [25,26], which in turn permits Mig1 to enter the nucleus [23]. There is, however, no evidence that Mig2 is controlled by Snf1, nor is its intracellular localization regulated by glucose [19]. Instead, the *MIG2 *gene is glucose induced [21]. It is likely that this, combined with glucose repression of *MIG1 *[19], is the reason why Mig2-dependent repression of the *HXT *genes is more prononuced at higher concentrations of glucose. Furthermore, we note that glucose induction of *MIG2 *expression can provide an explanation for the observation that *HXT6 *expression is regulated by Snf3 [54], since glucose induction depends on Snf3 [19].

In contrast to the extensive functional overlap between Mig1 and Mig2, we saw no evidence that Mig3 regulates the same set of genes as Mig1 and Mig2, at least not on 2% glucose. It has previously been reported that Mig3 contributes marginally to repression of some glucose repressed genes, *i. e. SUC2*, *HXT2*, *SNF3*, *MRK1 *and *MTH1 *[19,21], but only one of these, *MRK1*, was affected in our *mig3 *deletion. *MRK1 *is a protein kinase involved in stress signaling [55], which is interesting since Mig3 has been implicated in the response to genotoxic stress [29]. However, the most significant effect of a *mig3 *deletion (p-value 2.5e-3) in our microarrays was on the *SIR2 *gene, which was upregulated also in the *mig3 *double and triple mutants. Furthermore, this effect was found to be significant when verified by real-time PCR (Figure 6).

*SIR2 *encodes a histone deacetylase which is involved in silencing, but Sir2 has also been implicated in maintaining genome integrity [56], and in counteracting aging in both yeast and animals. A *sir2 *deletion reduces the replicative life span [57] but increases the chronological life span of yeast [58]. Little is known about regulation of *SIR2 *expression. Our finding that Mig3 downregulates *SIR2 *provides a possible explanation for the observation that reduced Snf1 activity leads to accelerated aging in yeast [59], since Snf1 inhibits Mig3 [29]. The latter effect was discovered within the context of the genotoxic stress pathway, and the fact that *SIR2 *is involved in the maintenance of genome integrity [56] would make it a logical target for that pathway. However, turnover of Mig3 was also regulated in a Snf1-dependent way by the carbon source [29], and the possibility therefore exists that Mig3-dependent regulation of *SIR2 *could respond to nutrient signaling pathways that are involved in the control of aging in yeast [60,61].

## Conclusion

Combinatorial gene regulation, where several transcription factors together regulate a set of target genes, enables cells to fine-tune their responses to different conditions. We have examined the role of three related yeast zinc finger proteins, Mig1, Mig2 and Mig3, which mediate signals from at least two signaling pathways (the Snf1 glucose repression pathway and the Rgt1 glucose induction pathway). To elucidate how the three proteins contribute to the control of gene expression, we used microarrays to study the expression of all yeast genes in the wild type and in all seven possible combinations of *mig1, mig2 *and *mig3 *deletions. Our data shows that Mig1 and Mig2 repress overlapping sets of genes, with Mig1 being the major repressor on 2% glucose, whereas Mig2 appears to be more important at higher glucose concentrations. Several genes that are redundantly repressed by Mig1 and Mig2 are involved in phosphate metabolism, and the promoters of these genes are enriched for a variant Mig1 site which is absent from genes repressed only by Mig1. Mig3 does not seem to target the same genes as Mig1 and Mig2. Instead, Mig3 represses the *SIR2 *gene encoding a histone deacetylase which is involved in gene silencing and the control of aging.

## Methods

### Yeast strains

The wild type strain used was BY4742. BY4742 congenic *mig1*, *mig2 *and *mig3 *single knockout mutants were obtained from the Euroscarf collection, and have the open reading frame of each gene replaced by the *KanMX *selection cassette. Congenic double and triple knockout mutants (see additional file 4: Table S3) were made by crosses followed by tetrad dissection.

### Microarray experiments

Three to five biological replicates were used for each strain. Cells were grown overnight at 30° on YPD with 2% glucose, diluted to A_600 _of 0.2, and then grown to an A_600 _of 1. The cells were pelleted by centrifugation and frozen in liquid nitrogen. A RiboPure™-Yeast kit (Ambion) was used for RNA preparation, after which the RNA was hybridized to Affymetrix YG_S98 GeneChip arrays. Raw data is available from Array Express [62] with accession number E-TABM-448.

### Microarray data analysis

The GCRMA pipeline [63] as implemented in the Bioconductor [64] library *gcrma *was used for background correction, normalization, and to summarize signals from individual probes in a probeset into one expression measure. Hypothesis testing, in the form of a linear method analysis [65,66] was then applied to extract contrasts between various pairs of strains. All contrasts differing in one specific deletion (e.g. between *mig1 mig3 *and *mig3*, and between *mig3 *and the wild type) were considered. For this, we used the Bioconductor package *Limma *with a rather conservative threshold: differences between strains were considered significant if the p-values for the moderated t-statistic were below 0.05 after FDR adjustment [67], and the magnitude of the expression difference was at least 2-fold. Only probe sets for ORFs, tRNA genes and genes from the mitochondrial genome were included in the analysis. Details about the linear models used are given in additional file 5: Additional documentation.

We also wanted to quantify to what extent Mig1 and Mig2 cooperate in regulating their target genes. Since Mig2 has very little effect on its own (see Results), we focused on the wild type, *mig1 *and *mig1 mig2 *strains. From expression measurements in these three strains we defined a redundancy measure, further explained in additional file 5: Additional documentation, as follows:

$$r=1-\left(\frac{4}{\pi }\right)\arctan\left(\frac{\log\text{ratio }\left(\text{mig1-wt}\right)}{\log\text{ratio }\left(\text{mig1mig2-wt}\right)}\right)$$

For finding enriched Gene Ontology annotations [68], we used the GO term finder [69]. Annotations were considered enriched if they had a p-value below 0.01 and were assigned to at least three genes.

### Promoter sequence analysis

Fisher's exact test was used to detect motifs enriched in the promoters of a given set of genes, as compared to all yeast genes. Motifs with p-values below 10^-5 ^were considered significantly enriched. Promoter regions (800 bp upstream of the transcription start site, or until the next ORF was reached) were taken from the RSAT database [70]. A list of *S. cerevisiae *motifs from T-profiler [71] were tested for enrichment, along with some additional motifs such as the complete Mig1 motif [15] and the PDS site [72]. In total, 152 motifs were tested (see additional file 6: Table S4). To find motifs that differ in frequency between different regulatory modes, the redundancy ratios of genes with and without each motif were compared using Wilcoxon's rank-sum test. We also wanted to find novel DNA motifs that could explain differences between the regulatory modes. For this, we used the BCRANK program with promoters of genes repressed by Mig1 and Mig2, ordered by redundancy. BCRANK is an open source R-package (available through Bioconductor, [64]) that takes a ranked list of sequences as input and outputs motifs that are overrepresented in one end of the list over the other. This is done using a heuristic search strategy starting from a randomly generated motif. For more details on analyses, see additional file 5: Additional documentation.

### Analysis of nucleosome occupancy

Genome wide ChIP-chip data on nucleosome occupancy were taken from [38]. Hybridization data was extracted around predicted Mig1 sites and STRE elements in all promoters, and averaged using a 20 bp wide sliding window. To test for differences in nucleosome occupancy around predicted binding sites between the two groups of genes (Mig1/2-repressed and not Mig1/2-repressed), we used Wilcoxon's rank sum test on the average hybridization levels spanning 50 bp around the motif.

### Real-time PCR

The real-time PCR analysis of *HXT *gene expression used three biological replicates for each condition, whereas the analysis of *SIR2 *expression used five replicates. Cells were grown at 30°C in YPD with either 2% or 10% glucose. To ensure that all cells were in log phase, cultures were serially diluted for 24 hours, keeping A_600 _between 0.1 and 1.0, after which the cells were diluted to an A_600 _of 0.2 and grown until an A_600 _of 1. Harvesting of cells and extraction of RNA were performed as in the microarray experiment, after which the RNA samples were treated with DNAse I (Ambion), and used for reverse transcription. Reverse transcription was performed using RevertAid™ H Minus M-MuLV Reverse Transcriptase (Fermentas), according to the manufacturer's instructions. The PCR reactions were carried out on an ABI prism detector 7700 (Applied Biosystems), using TaqMan Gold polymerase (Applied Biosystems), and the primers and probes listed in additional file 7: Table S5.

## Authors' contributions

JOW and EM grew yeast strains and prepared RNA for the array experiments. JOW did the bioinformatic analysis of the data and drafted the manuscript. NN and JOW did the real-time PCR experiments. AA did some of the statistical work. JK and HR conceived of the study and coordinated the work. All authors read and approved the final manuscript.

## Supplementary Material

Additional file 1 **Table S1.** Genes that are downregulated by Mig1 and/or Mig2. The file contains a list of genes that are downregulated by Mig1 and/or Mig2. For each gene the following is shown: ORF name, gene name, functional annotation, relative expression compared to the wild type strain after deletion of either the *MIG1 *gene alone (*mig1*) or *MIG1 *and *MIG2 *(*mig1 mig2*), p-values for these changes, redundancy measure, and whether the promoters contain Mig1 binding sites or not. Click here for file

Additional file 2 **Table S2.** Genes that are upregulated by Mig1 and/or Mig2. The file contains a list of genes that are upregulated by Mig1 and/or Mig2. For each gene the following is shown: ORF name, gene name, functional annotation, relative expression compared to the wild type strain after deletion of either the *MIG1 *gene alone (*mig1*) or *MIG1 *and *MIG2 *(*mig1 mig2*), p-values for these changes, redundancy measure, and whether the promoters contain Mig1 binding sites or not. Click here for file

Additional file 3 **Figure S1.** Microarray data for hexose transporter gene expression The file contains a figure showing the microarray expression data of the hexose transporter genes *HXT1*, *HXT2*, *HXT3*, *HXT4 *and *HXT6/7*. Click here for file

Additional file 4 **Table S3.** Yeast strains. The file contains information about the yeast strains used in the present study. Click here for file

Additional file 5 **Additional documentation.** The file contains details about the linear model and redundancy measure used, and statistical support for different possible mechanisms for differences in specificity between Mig1 and Mig2. Click here for file

Additional file 6 **Table S4.** DNA sequence motifs. The file contains information about the DNA sequence motifs used in the present study. For each motif the following is shown: the sequecnce in IUPAC code, the name of the motif, and the source as listed in T-profiler [71]. Click here for file

Additional file 7 **Table S5.** Oligonucleotide primers used for real-time rt-PCR. The file contains information about the real-time rt-PCR oligonucleotide primers used in the present study. Click here for file
