# Supplementary figures and images for: Combinatorial control of gene expression by the three yeast repressors Mig1, Mig2 and Mig3
Source: BMC Genomics. 2008 Dec 16;9:601. doi: 10.1186/1471-2164-9-601 (PMC2631581; doi:10.1186/1471-2164-9-601)

**Figure S1: Microarray data for hexose transporters**

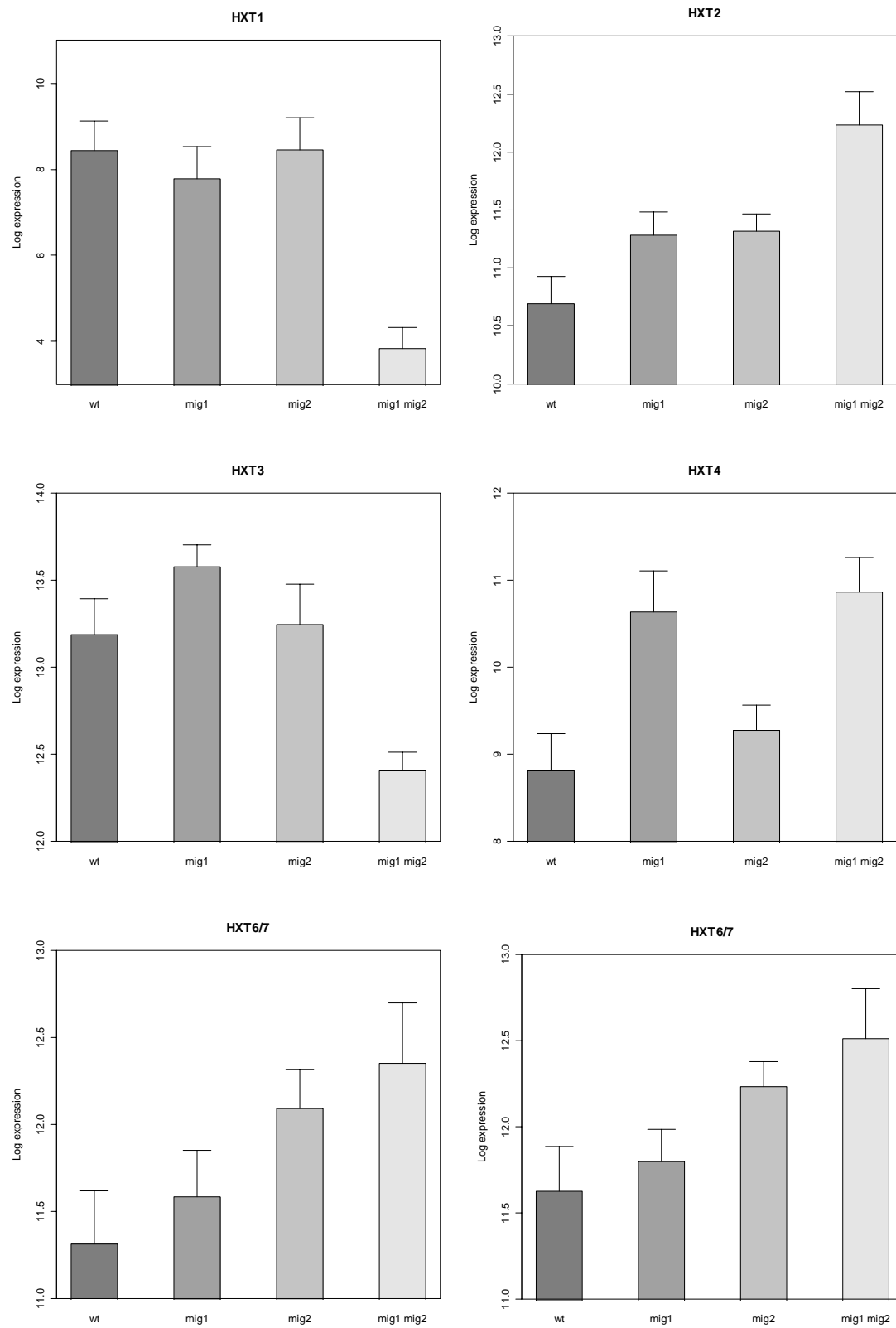

Supplement: Additional file 3 — Figure S1. Microarray data for hexose transporter gene expression The file contains a figure showing the microarray expression data of the hexose transporter genes HXT1, HXT2, HXT3, HXT4 and HXT6/7. [file 1471-2164-9-601-S3.pdf]
