# Supplementary material for: Combinatorial control of gene expression by the three yeast repressors Mig1, Mig2 and Mig3
Source: BMC Genomics. 2008 Dec 16;9:601. doi: 10.1186/1471-2164-9-601 (PMC2631581; doi:10.1186/1471-2164-9-601)
Supplement: Additional file 5 — Additional documentation. The file contains details about the linear model and redundancy measure used, and statistical support for different possible mechanisms for differences in specificity between Mig1 and Mig2. [file 1471-2164-9-601-S5.pdf]

Combinatorial control of gene expression by the three yeast repressors Mig1, Mig2 and Mig3 – Additional documentation

Details about the linear model

The linear model used to detect differentially expressed genes contains the factors *strain*, (with eight levels: *wt*, *mig1*, *mig2, mig3, mig1 mig2, mig1 mig3, mig2 mig3* and *mig1 mig2 mig3*) and a block factor to correct for the fact that RNA was prepared on different occasions. The model we used was thus

$$y = \alpha_{strain} + \beta_{block} + \varepsilon$$

where y is the measured gene expression level, α and β are the strain and block effects respectively, and ε are the residuals.

Details about the redundancy measure

Since we were interested in the relative contributions of Mig1 and Mig2 in regulation of the target genes, we defined a measure that explicitly quantifies this effect:

$$r = 1 - \left(\frac{4}{\pi}\right) \arctan\left(\frac{\text{log ratio}\,(\text{mig1 - wt})}{\text{log ratio}\,(\text{mig1mig2 - wt})}\right)$$

The rationale for considering the two log ratios of the contrasts *mig1*-wt and *mig1mig2*-wt is that we want to compare the contributions of Mig1 and Mig2 (considering only the Mig2 effect, for instance in the contrast *mig1mig2-mig1*, would not enable us to relate this effect to the contribution of Mig1). These two log ratios comprise a two-dimensional vector, and *arctan* gives the direction of this vector (see the plot below). For convenience, the direction is then rescaled with the factor (4/π) after which the scale is reversed, so that *r = 0* (blue line) corresponds to no contribution of Mig2 and *r = 1* (red line) to an equal contribution of Mig1 and Mig2.

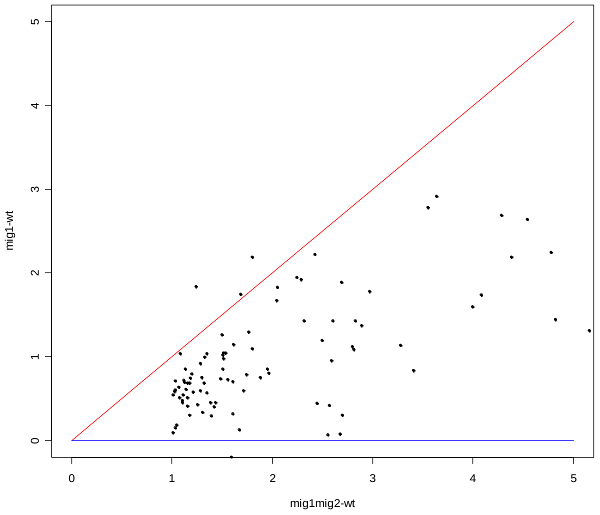

An alternative to using the measure defined above would be to cluster the genes. For instance, clustering the genes on the log ratios *mig1*-wt and *mig1mig2*-wt with Pearson correlation as distance metric gave similar results to tables S1 and S2. However, since we were interested in directly quantifying the contribution of Mig2 in relation to Mig1, an explicit measure for this effect was motivated. This also enabled us to search for motifs with skew distributions of the redundancy measure.

Support for different mechanisms for Mig1/Mig2 specificity

Hypothesis b: Mismatches within the AT box

Some results in (Lutfiyya et al., Mol Cell Biol, 16(9), 4790-4797, 1998) suggested that Mig1 sites bound by Mig2 might have more mismatches (C or G) in the flanking AT box than Mig1 sites bound by Mig1. We therefore checked the correlation between the number of mismatches in the AT box and the redundancy ratio.

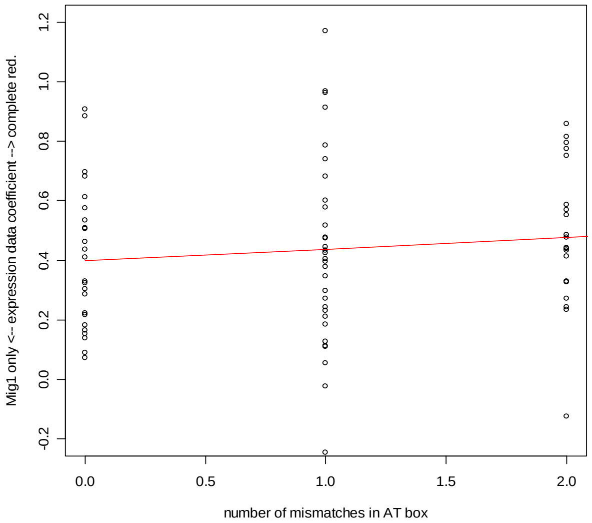

As this plot shows, the correlation between the number of mismatches in the AT box and the redundancy ratio is low (0.11), and the number of mismatches in the AT box is in fact not a significant (p-value=0.35) parameter for determining the redundancy ratio. Thus, we find no clear support for the hypothesis that the number of mismatches in the flanking AT box is important for the Mig1/Mig2 specificity.

Hypothesis c: Differences in positions of the site within the promoters

Next, we tested if the location of the Mig1 sites within the promoters could explain the different redundancy scores. Below is a plot of redundancy vs the distance from the (closest) Mig1 site upstream of the reading frame to the start codon.

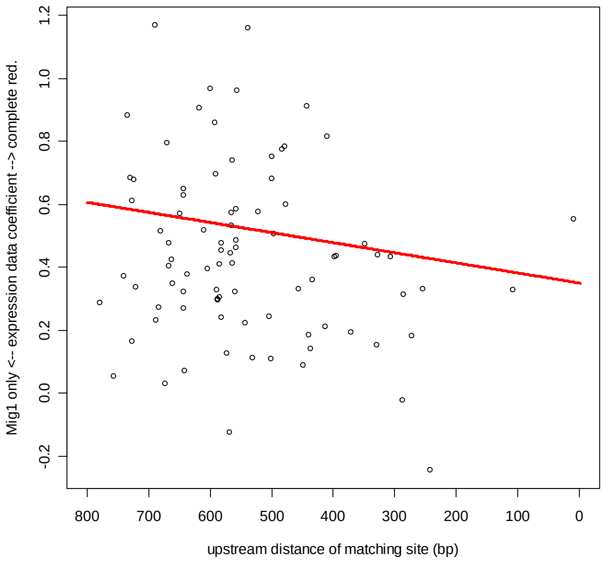

The correlation between the redundancy ratio and the position of the closest Mig site is -0.17 and the position of the Mig sites is not a significant (p-value=0.11) parameter for determining the redundancy ratio. We conclude that binding site location does not seem to be important for Mig1/Mig2 specificity.

Hypothesis d: Differences in orientation

The orientation of the Mig1 site is another possible mechanism behind the Mig1/Mig2 specificity. We therefore checked if there were any significant differences in orientation between the Mig1 sites in the promoters of the three groups of genes, using hypergeometrical tests against the background of all genes repressed by Mig1/Mig2. As the table below shows, neither orientation of the Mig1 motif was enriched in any group of genes.

	P-value	pos %	neg %
Total redundancy	0.38	41	59
Partial redundancy	0.62	45	55
Only Mig1	0.65	45	55

Hypothesis e: Differences in number of Mig1 sites between promoters

We also tested if the number of Mig1 sites could explain the difference in redundancy. The plot below shows redundancy ratios vs number of Mig1 sites in the promoter region.

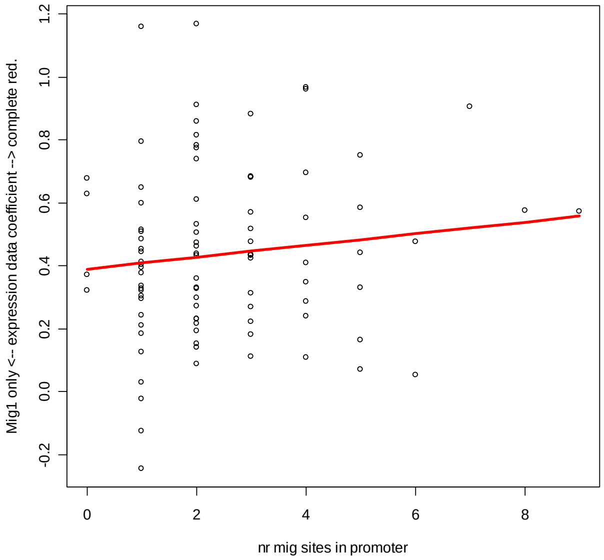

The correlation between the redundancy ratio and the number of Mig sites is low (0.12) and the number of Mig sites is not a significant (p-value=0.26) parameter for determining the redundancy ratio. Thus, the number of Mig1 sites cannot explain the differences in redundancy.
